# Changes in Biochemical Parameters of the Calcium-Phosphorus Homeostasis in Relation to Nutritional Intake in Very-Low-Birth-Weight Infants

**DOI:** 10.3390/nu8120764

**Published:** 2016-11-29

**Authors:** Viola Christmann, Charlotte J. W. Gradussen, Michelle N. Körnmann, Nel Roeleveld, Johannes B. van Goudoever, Arno F. J. van Heijst

**Affiliations:** 1 Department of Paediatrics, Subdivision of Neonatology, Radboudumc Amalia Children’s Hospital, Radboud University Medical Center, Nijmegen 6500HB, The Netherlands; charlotte.gradussen@radboudumc.nl (C.J.W.G.); michelle.kornmann@radboudumc.nl (M.N.K.); arno.vanheijst@radboudumc.nl (A.F.J.v.H.); 2 Department for Health Evidence, Radboud Institute for Health Science, Radboud University Medical Center, Nijmegen 6500HB, The Netherlands; nel.roeleveld@radboudumc.nl; 3 Department of Paediatrics, Radboudumc Amalia Children’s Hospital, Radboud University Medical Center, Nijmegen 6500HB, The Netherlands; 4 Department of Paediatrics, VU university medical center Amsterdam, Amsterdam 1081HV, The Netherlands; h.vangoudoever@vumc.nl; 5 Department of Paediatrics, Emma Children’s Hospital-AMC Amsterdam, Amsterdam 1105AZ, The Netherlands

**Keywords:** blood, bone mineralization, minerals, monitoring, nutrition, renal tubular reabsorption, supplementation, urine

## Abstract

Preterm infants are at significant risk to develop reduced bone mineralization based on inadequate supply of calcium and phosphorus (Ca-P). Biochemical parameters can be used to evaluate the nutritional intake. The direct effect of nutritional intake on changes in biochemical parameters has not been studied. Our objective was to evaluate the effect of Ca-P supplementation on biochemical markers as serum (s)/urinary (u) Ca and P; alkaline phosphatase (ALP); tubular reabsorption of P (TrP); and urinary ratios for Ca/creatinin (creat) and P/creatinin in Very-Low-Birth-Weight infants on Postnatal Days 1, 3, 5, 7, 10, and 14. This observational study compared two groups with High (*n* = 30) and Low (*n* = 40) intake of Ca-P. Birth weight: median (IRQ) 948 (772–1225) vs. 939 (776–1163) grams; and gestational age: 28.2 (26.5–29.6) vs. 27.8 (26.1–29.4) weeks. Daily median concentrations of biochemical parameter were not different between the groups but linear regression mixed model analyses showed that Ca intake increased the uCa and TrP (*p* = 0.04) and decreased ALP (*p* = 0.00). Phosphorus intake increased sP, uP and uP/creat ratio and ALP (*p* ≤ 0.02) and caused decrease in TrP (*p* = 0.00). Protein intake decreased sP (*p* = 0.000), while low gestational age and male gender increased renal excretion of P (*p* < 0.03). Standardized repeated measurements showed that biochemical parameters were affected by nutritional intake, gestational age and gender.

## 1. Introduction

Bone development is one of the key processes of intrauterine and postnatal growth [1]. Preterm infants are at significant risk to develop reduced bone mineral content based on inadequate supply of calcium and phosphorus (Ca-P) [2,3]. During normal pregnancies in healthy mothers, there is an active, placental transfer of Ca-P to the fetus leading to a high mineral accretion during the last trimester, while after birth the infant is dependent on nutritional supply of minerals [4,5]. In clinical practice, postnatally it is difficult to meet the high fetal needs, because of limited solubility of parenteral fluids, low content of Ca-P of human milk and impaired intestinal absorption through formula feeding [6,7,8,9]. Studies tried to define nutritional requirements, but, in clinical practice, it is often uncertain whether the nutritional intake of Ca-P provided to preterm infants is sufficient and is actually used for bone mineralization [10,11,12].

Assuming that biochemical parameters of Ca-P homeostasis within a normal range will lead to optimal bone mineralization, evaluation of electrolyte disturbances is standard of care in many neonatal units [13,14,15,16,17,18,19,20,21,22,23,24,25,26,27,28]. However, there is currently neither a consensus on the appropriateness of either parameter or the frequency of measurements [29,30]. A recent survey among U.S. neonatologists showed a great lack of consensus and variation in practices regarding definition and screening methods for metabolic bone disease [31]. Reference values in relation to adequate nutritional intake have not been developed. Urinary excretion of minerals in spot urine samples has been shown to be an easy tool for routine evaluation. Pohlandt proposed to aim for a small “surplus of minerals” in urine samples, while Aladangady et al. developed reference values for urinary Ca-P/creatinine ratios for preterm infants [17,23]. Staub et al. compared both methods with regard to an agreement between their results and found neither method to be superior [32]. None of the studies evaluated the direct effect of nutritional intake on biochemical parameter of Ca-P homeostasis. It is not sure whether biochemical parameters are able to indicate sufficiency of nutritional intake.

The aim of this study was to evaluate changes in biochemical parameters of the Ca-P homeostasis in blood and urine in relation to different nutritional intake during the first 14 days of life in Very Low Birth Weight (VLBW) infants. Our hypothesis was that the nutritional intake of calcium and phosphorus would have an effect on biochemical parameters of the calcium–phosphorus homeostasis.

## 2. Materials and Methods

### 2.1. Study Design and Randomization

The current study (Early Supplementation Study (ESS)) was part of the Early Nutrition Study (ENS), a multi-center double-blinded randomized controlled trial.While the ENS evaluated the effects of human milk on postnatal outcome, the primary objective of the ESS was bone mineralization in relation to early and late enteral supplementation of minerals [33]. The studies were approved by the Ethical Committee of the VU university medical center (Amsterdam, The Netherlands), 13 September 2013 (CMO file number: NL37296.029.11, Dutch Trial Registry: NTR 3225). Patients were distributed into three groups through two steps of randomization. The first step randomized eligible infants either into the early supplementation group (High) or the late supplementation group (Low) being part of the ENS. The second step of the randomization was only performed if infants were assigned to late supplementation and randomized them to either “ENS A” or “ENS B” as part of the ENS. Both randomization steps were performed before the first enteral nutrition was administered resulting in basically three groups.

### 2.2. Study Population

Participants for the ENS/ESS were recruited at the level III neonatal intensive care unit of the Radboud university medical center (Radboudumc), Nijmegen, The Netherlands. The inclusion criteria were a birth weight below 1500 grams and written informed consent of both parents. The exclusion criteria were maternal drugs and/or alcohol use during pregnancy, birth defects, congenital infection within 72 h after birth, perinatal asphyxia with a pH < 7.0 and any intake of cow’s milk based products prior to randomization. For the current study, infants who died or were discharged before the end of the study period of 14 days were excluded from analysis.

### 2.3. Intervention and Nutritional Protocol

Parenteral nutrition (PN) was started directly within the first hour after birth. The PN solution consisted of 2.5 mmol/dL calcium-gluconate (calcium-gluconate 10%; B. Braun, Melsungen, Germany) and 1.6 mmol/dL sodium-glycerophosphate (Glycophos; Fresenius Kabi BV, Zeist, The Netherlands) and 2.25 grams/dL amino acids (Primene; Clintec, Brussels). Additional parenteral supplementation with 10% calcium-gluconate or sodium glycero-phosphate was administrated depending on biochemical parameters. Table A1 presents the standard protocol for PN. The decision to start additional supplementation was left to the attending neonatologist based on biochemical parameter as being a standard procedure of our department.

Enteral feeding was started on the first day of life with daily increments, while PN was gradually reduced to maintain a daily fluid intake within the protocol range. Late supplementation was assumed to provide a low intake of nutrients because “Group Low”, comprising of group ENS A and ENS B, received no additional enteral supplementation or fortification of human milk during the first 10 days of life. Group ENS A received donor milk if mother’s own milk (MOM) was not available. Group ENS B received preterm formula (Hero Baby Prematuur Start; Hero Kindervoeding, Breda, The Netherlands) if MOM was not available, containing 2.4 mmol/dL calcium, 1.7 mmol/dL phosphorus and 2.6 grams/dL proteins. The additional nutrition in ENS A and ENS B was blinded to all caretakers and parents. After 10 days, all infants received nutrition according to the standard protocol of the Radboudumc.

Early supplementation was assumed to provide a high intake (Group High). This group received enteral nutrition from Day 1 onwards according to the local protocol. They received additional enteral supplementation and human milk fortifier (Nutrilon Neonatal BMF; Nutricia, Zoetermeer, The Netherlands; BMF) by the time the enteral intake was 50 mL per day. The human milk fortifier added 1.65 mmol/dL calcium, 1.22 mmol/dL phosphorus and 0.8 grams/dL protein to human milk. They received preterm formula if MOM was not available. The decision to start additional enteral supplementation was left to the attending neonatologist based on biochemical parameter and postnatal growth as being a standard procedure of our department. The additional enteral supplementation could comprise of either a supplement of protein (Nutrilon Nenatal Protein Fortifier; Nutricia, Zoetermeer, The Netherlands) or a potassium phosphate (KPO_4_) and calcium chloride (CaCl_2_) suspension for enteral supplementation.

Group ENS A received 100% human milk during the first 10 days and reflected a group with low intake of minerals and protein, because human milk has a very low nutrient content. Group High reflected a high intake of nutrients, because human milk was enriched with minerals and protein as soon as possible. Group ENS B could be considered as intermediate depending on the amount of MOM or preterm formula an infant received, since preterm formula contained approximately the same amount of minerals as fortified human milk.

### 2.4. Biochemical Parameters of Bone Mineralization

For this study, blood and urine samples were analyzed according to the local protocol of the department. Samples were taken on Days 1, 3, 5, 7, 10 and 14 after birth. Urine was collected through spot samples [25]. The following parameters were analyzed: serum calcium (sCa), serum phosphorus (sP), serum alkaline phosphatase (ALP), urine calcium (uCa), urine phosphorus (uP), urine calcium/creatinin ratio (uCa/Creat), urine phosphorus/creatinin ratio (uP/Creat), and tubular reabsorption of phosphorus (TrP).

### 2.5. Data Registration and Handling

Patient characteristics, clinical course, growth and intake of all nutrients were recorded daily from the patient records and abstracted for this study. Amounts of enteral, parenteral and additional supplementation (parenteral and enteral) of all nutrients were calculated separately for each patient. The total intakes were calculated per kg per day per infant. The intake through human milk was calculated based on the reference of Gidrewicz et al. [34]. The calcium/phosphorus ratio was calculated per day by dividing the daily intake of calcium in mmol/kg through the daily intake of phosphorus in mmol/kg.

After closure of patient enrollment and de-blinding of the ENS, we performed a reallocation procedure for the intermediate group ENS B. Infants who received more than 90% MOM were considered to reflect a low intake of minerals and were allocated to group Low together with the infants of group ENS A. Infants who received more than 90% of preterm formula were considered to reflect a high intake of minerals and were allocated to High. Infants in between these extremes were not included in the analyses.

### 2.6. Statistical Analysis

The primary objective of the ESS was bone mineralization in relation to mineral supplementation, and the original power calculation was based on bone mineral content at term corrected age. For the evaluation of changes in biochemical parameters in relation to nutritional intake the power calculation was based on sP. In a previous evaluation of our nutritional protocol performed at our department, we found a mean 1.7 mmol/L of sP during the first week [35]. A concentration of 2.0 mmol/L was defined as target for optimal bone mineralization by Hellstern et al. [20]. Assuming an expected mean of 1.7 mmol/L, we determined that 24 infants were required in each group to find a difference of 0.3 mmol/L in sP between High and Low with α = 0.05 (two-sided) and a power of β = 0.80.

The statistical analyses were performed using IBM SPSS statistics 22.0 for Windows (IBM SPSS Inc., Chicago, IL, USA). Differences in patient characteristics, nutritional characteristics and biochemical parameters between the High and Low group were determined using the Mann-Whitney U test or the chi-square test, depending on the variable under examination. Due to non-normality of the continuous variables, the data were presented as median (with interquartile range (IQR)), unless otherwise indicated. A *p*-value < 0.05 was considered statistically significant.

To account for repeated outcome measurements, we used a mixed model analysis to determine the effects of daily nutritional intake of calcium and phosphorus on each biochemical parameter. We included the total intake of Ca/P and protein, the percentage of enteral amount of Ca/P intake, and a number of clinical parameter that could affect the Ca/P homeostasis such as birth weight, gestational age, gender, caesarian section, multiple births, sepsis, and days of caffeine, furosemide, steroids, and sedation during the first two weeks as co-variables in the initial models. Necrotizing enterocolitis was not included as co-variable because of small numbers. Using manual backward selection, variables were kept in the model when they contributed statistically significantly with a *p*-value < 0.1.

## 3. Results

### 3.1. Patient Characteristics

**Figure 1:**
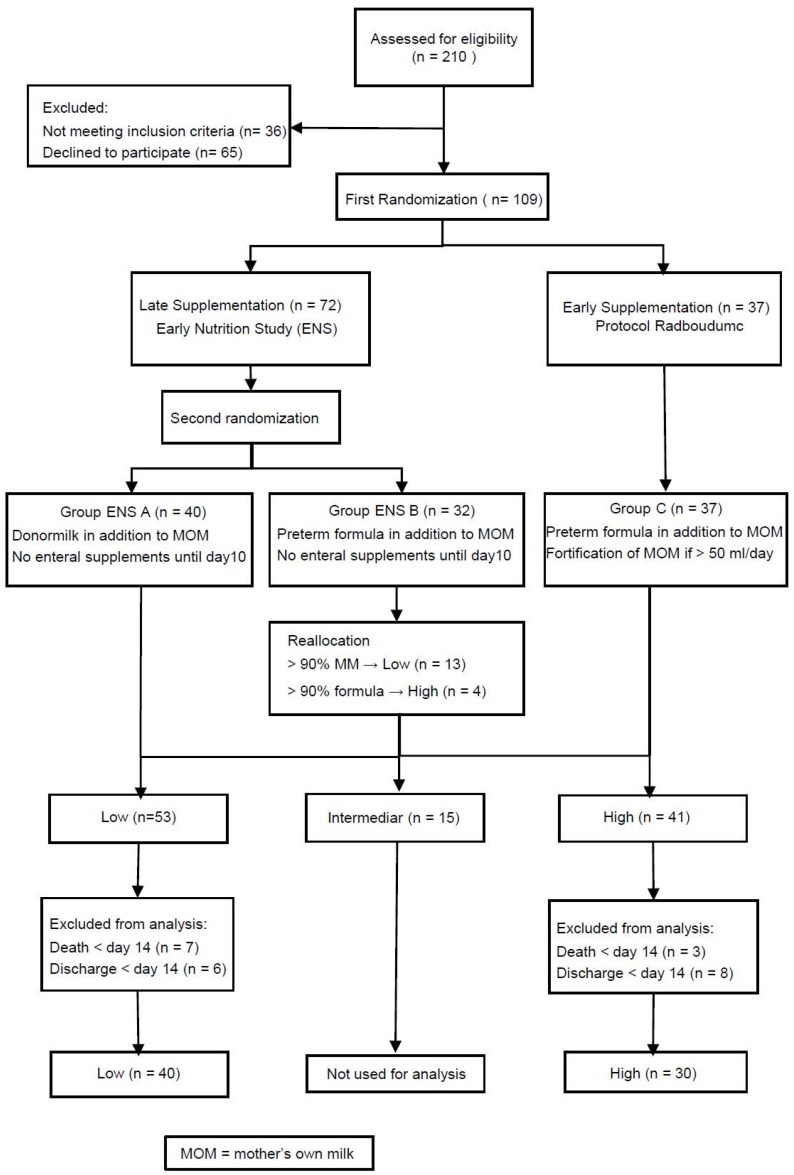
Consort diagram.

Enrollment of patients occurred between January 2013 and December 2014. The distribution of the infants is presented in the consort diagram (Figure 1). Finally, 109 infants were randomized, either to Late Supplementation (Low; *n* = 72; distributed into Group ENS A (*n* = 40) and ENS B (*n* = 32) or Early Supplementation (High; *n* = 37).

The characteristics of all infants included in the three groups of the ENS/ESS study are presented in Table A2. After de-blinding of group Low, four infants of group ENS B were reallocated to High and 13 to Low so that Low and High consisted of 53 and 41 infants, respectively. Infants who died or were discharged before postnatal Day 14 (13 in Low, 11 in High) were excluded. Finally, data of 40 infants of Low and 30 of High were analyzed. The baseline patient characteristics, morbidity, medication and nutritional characteristics for these patients are presented in Table 1. Infant characteristics were well balanced between the groups Low and High and comparable to the original groups.

### 3.2. Nutritional Intake

The nutritional characteristics and intake of calcium, phosphorus and protein during Weeks 1 and 2 are presented in Table 1. The median and interquartile range (IQR) for the duration of PN was 12.0 (10.0–14.0) versus 11.0 (9.0–14.0) days for High versus Low, while the median day of reaching an enteral intake of 150 mL/kg was Day 13.0 (10.5–20.0) versus Day 12.0 (9.8–17.0), respectively. In accordance with the study protocol, Low received a higher amount of human milk. The median start day of BMF in groups High and Low was 7.9 (5.0–10.0) and 11.0 (11.0–13.0) respectively. As a result, High received a significant higher total intake of calcium and phosphorus during the first two weeks and of protein during Week 2 compared to Low. Table A3 presents the nutritional intake divided into four routes of administrations: parenteral, enteral and additional supplementation either par- or enteral. This shows that differences in intake were mainly based on differences in enteral intake. Further, both groups received additional parenteral supplementation of phosphorus, based on low sP concentrations.

**Figure 2:**
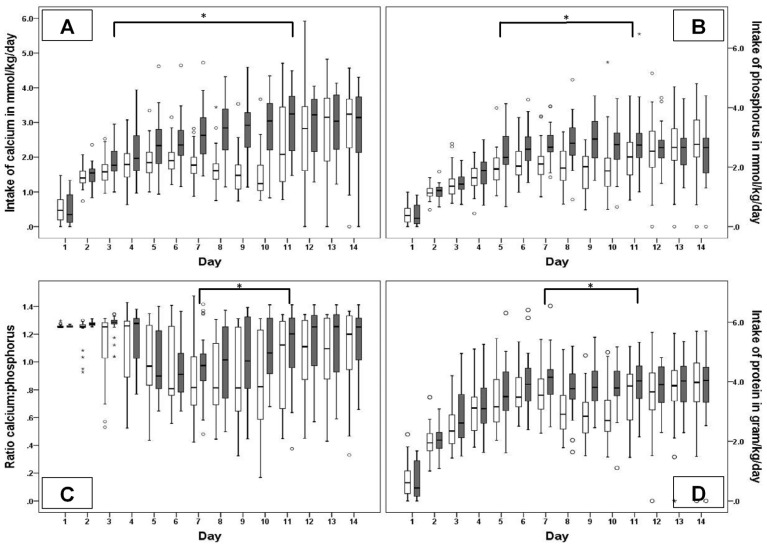
Nutritional intake during the first 14 days: white bars: Low; black bars: High; The horizontal black bars with * indicate the days on which the intake was statistically significant different. (**A**) Daily intake of calcium in mmol/kg/day; * = *p* < 0.05; (**B**) daily intake of phosphorus in mmol/kg/day; * = *p* < 0.05; (**C**) daily intake of protein in gram/kg/day; * = *p* < 0.05; and (**D**) ratio calcium intake to phosphorus intake; * = *p* < 0.05 Data are presented as median, interquartile range and upper and lower limits.

Figure 2 presents the daily changes in nutritional intake during the first 14 days. Figure 2A,B,D demonstrate the total calcium, phosphorus and protein intake. High had a steady increase in intake during the study period, whereas Low showed a temporary decrease, and plateau at the end of the observational period, probably due to the decreasing amount of PN and increasing amount of unfortified human milk. Except for Day 1, both groups received a median total calcium, phosphorus and protein intake according to the ESPGHAN recommendations for parenteral and enteral nutrition [38,39]. The calcium/phosphorus ratios were highly variable. Both groups showed a decrease in the calcium/phosphorus ratio on Day 5 that lasted until Day 11 (Figure 2C), most likely caused by the transition from parenteral nutrition to enteral nutrition. For both groups, the ratio was below the recommendations of ESPGHAN on all days [38,39].

### 3.3. Biochemical Parameters

Table A4 summarizes the median daily values of both groups for all biochemical parameters. The median serum concentrations of Ca en P were within the normal range and only showed slight differences between the two groups and an overall increase during the study period [40]. Except for the first day, the median sP concentrations remained below our target of 2 mmol/L until Days 5 and 10 for High and Low, respectively. The median uCa and uP values were above the recommended surplus (uCa > 1.2 mmol/L, uP > 0.4 mmol/L) during the entire observational period [23]. The median TrP values were above the lower normal range of 85% until Day 5, and decreased thereafter, reflecting a higher loss of phosphorus. The median ALP values were within the normal range (80–330 U/L) until Day 5, but increased steadily thereafter [40]. In both groups, the uCa/Creat ratios were above the reference value (0.5 mmol/mmol) during the complete study period [32]. The uP/Creat ratios were below the reference value (4.0 mmol/mmol) until Day 5, but above the reference thereafter [32].

The results of the mixed model analyses are summarized in Table 2.

- The sCa concentration was not related to intake of Ca/P and was only marginally affected by a number of co-variables except for daily protein intake that caused an increase of 0.107 mmol/L per gram/kg protein.
- The sP concentration increased in relation to phosphorus intake (0.13 mmol/L per mmol/kg phosphorus) and birth weight (0.0004 mmol/L per gram birth weight), whereas protein intake (−0.13 mmol/L per gram/kg/day protein), gestational age (−0.05 mmol/L per week), furosemide (−0.11 mmol/L per day) and caffeine (−0.02 mmol/L per day) decreased in sP concentration.
- The urinary excretion of Ca seemed to increase in relation to Calcium intake (0.35 mmol/L per mmol/kg calcium), and increased in relation to protein (0.36 mmol/L per gram/kg protein) and being born by cesarean section (0.65 mmol/L if born by cesarean section), whereas it was not affected by the phosphorus intake.
- The urinary excretion of P increased in relation to daily phosphorus intake (3.18 mmol/L per mmol/kg phosphorus) and gender (1.88 mmol/L if infant was a boy), whereas P excretion lowered in relation to daily intake of protein (−1.18 mmol/L per mmol/kg protein), gestational age (−0.71 mmol/L per week) and caffeine (−0.29 mmol/L per day). Calcium intake did not affect the urinary P excretion.
- The TrP increased in relation the daily Calcium intake (3.10% per mmol/kg calcium) and gestational age (3.05% per week). The reabsorption of phosphorus lowered in relation to daily phosphorus intake (−6.21% per mmol/kg phosphorus), gender (−4.60% if infant was a boy), being born by cesarean section (−5.12%), and sepsis (−6.78%).
- The ALP increased in relation protein intake (30.54 U/L per mmol/kg) and daily intake of phosphorus (23.64 U/L per mmol/kg phosphorus). A decrease in ALP was related to calcium intake (−44.94 U/L per mmol/kg calcium), gestational age (−20.71 U/L per week) and the number of days of steroid use (−23.86 U/L per day).
- The uCa/creat ratio increased in relation to daily protein intake (0.54 L/L per gram/day protein) and sepsis (0.66 L/L), but it was not affected by the total calcium and phosphorus intake.
- The uP/creat ratio increased in relation to daily phosphorus intake (4.01 L/L per mmol/kg phosphorus), gender (2.31 L/L if infant was a boy), while the P/creat ratio seemed lower in relation to daily protein intake (−0.81 L/L per gram/kg protein), and decreased with gestational age (−0.94 L/L per week), and caffeine (−0.30 L/L per day).

## 4. Discussion

In this observational study of initially three randomized groups providing different nutritional intake to VLBW infants during the first 10 days of life, we found no differences between groups Low and High concerning the biochemical parameters of Ca-P homeostasis. However, the mixed model analysis showed that the intake of calcium was associated with increased urinary calcium excretion and tubular reabsorption of phosphorus and a decrease in the ALP, while the nutritional intake of phosphorus was associated with a decreased sCa and an increase in sP, uP and uP/creat ratio. The nutritional intake of calcium and phosphorus affected the TrP and ALP in opposite directions. Protein intake was greatly associated with a decrease in sP, uP and an increase in ALP, sCa, and uCa, while, in addition, gestational age and male gender affected especially the phosphorus metabolism.

VLBW infants belong to one of the most vulnerable patient groups for whom adequate postnatal nutritional intake has life-long consequences [41]. Therefore, intervention studies with different nutritional intakes could be seen as unethical in the light of the right of optimal treatment for every patient. On the other hand, in clinical practice a great variation in clinical guidelines has been reported, often based on rather low evidence [42]. While fortification of human milk is generally seen as necessary nowadays, there is also concern about possible risks of introducing cow-milk based products too early [43,44]. According to our local protocol, fortification is introduced early and additional mineral supplementation is provided based on laboratory results. The intention is to optimize postnatal growth and bone mineralization but the efficacy of our protocol has not been proven. The combination of the Early Nutrition Study and the Early Supplementation Study provided the opportunity to evaluate two different nutritional concepts within the range of nutritional guidelines and therefore within the ethical limits. On the other hand, all infants participating in the ESS, independent of group allocation, received the standard treatment according to the local practice, which frequently led to additional parenteral supplementation of nutrients in case of electrolyte disturbances or impaired growth. This practice may have ameliorated the differences between the groups and therefore affected the results. By reallocating infants from group ENS B to either group Low or High and excluding infants with intermediate intake from further analysis, we tried to maximize the differences in nutritional intake between the two remaining groups. The reallocation of infants did not change the baseline patient characteristics. The detailed analysis of nutritional intake showed that additional supplementation was not different between the groups and differences in intake were mainly based on enteral nutrition.

Even though the two groups had a maximum difference in nutritional intake, the comparison of daily concentrations of the biochemical parameters showed no differences between groups High and Low, probably by leveling out inter-individual differences on group level. In contrast, the linear mixed model analysis took into account both intra- and inter-individual fluctuations, and thereby enabled us to specify effects of various co-variables.

Despite an increasing intake of phosphorus, sP remained below our target concentration during the first week. Recently, a randomized trial, evaluating nutritional support according to current recommendations in VLBW infants, observed hypophosphatemia in relation to high protein intake [45]. Jamin et al. observed electrolyte disturbances, especially hypophosphatemia and hypokalemia, in low-birth weight piglets with a high protein diet [46]. Hypophosphatemia is the hallmark of the refeeding syndrome and a well-known complication in relation to parenteral nutrition of malnourished patients [47,48,49]. Bonsante et al. proposed the concept of Placental Incompletely Restored Feeding (PI-Refeeding) syndrome for electrolyte disturbances found in VLBW infants [50]. This syndrome is said to be caused by an imbalanced nutritional intake of amino acids and phosphorus. Amino acids and energy are needed to maintain an anabolic state of the cell, while phosphorus is necessary for a number of cellular functions, energy homeostasis as well as for bone mineralization. Phosphorus in blood will preferably be transferred to the cell regardless of bone mineral status. A higher intake of amino acids will enhance the need for phosphorus in growing cells, and in case of low concentrations of phosphorus in blood it will be released from bone. Simultaneously with the release of phosphorus, calcium will also be released from the bone because of an unfavorable Ca/P ratio and will consecutively be excreted in urine if the sP concentrations are too low. Our results are in agreement with this concept. According to the mixed model analyses, we found that an increasing amount of protein was associated with an increase in the sCa, uCa, ALP and uCa/Creat ratio, whereas it was associated with a decrease in sP, uP and the uP/Creat ratio. Remarkably, in our study, an increase in a sP concentration of 0.13 mmol/L occurred per 1 mmol/kg intake of phosphorus and a decrease of −0.13 mmol/L per 1 gram/kg protein intake, meaning that 1 gram/kg of protein intake should be accompanied by 1 mmol/kg of phosphorus in nutrition of VLBW infants to maintain adequate sP concentrations.

The role of ALP in bone mineralization is controversial, but an increase is usually associated with poor bone mineralization [30,51]. According to our results, an increasing intake of protein was associated with an increase in ALP. Again, following the above mentioned mechanisms higher protein intake enhanced the cellular need of phosphorus and thereby decreased the sP concentration and the availability of phosphorus for bone mineralization, leading to activation of ALP. We also found that an increased ALP was associated with increasing phosphorus intake, while one would expect lowering of ALP. An explanation for this phenomenon could be a relatively insufficient intake of calcium in combination with phosphorus intake, since an increasing calcium intake was associated with decrease in ALP concentrations. In this study, for both groups, the calcium/phosphorus ratio was below recommendations, meaning that relatively more phosphorus than calcium was administered.

Gestational age at birth seemed to be an important determinant for the phosphorus metabolism in our study, meaning that infants with a lower gestational age had a higher renal excretion of phosphorus, irrespective of nutritional intake. Immaturity of the kidneys at lower gestational age has been shown to cause impaired tubular reabsorption of phosphorus [15]. Renal losses of minerals may then compromise the effect of nutritional intake on bone mineralization. However, current recommendations for nutritional intake of calcium and phosphorus usually do not take into account differences in renal function based on gestational age.

Further, we found that male gender was related to low serum phosphorus concentrations, low tubular reabsorption and increased renal excretion of phosphorus and uP/creat ratio. We speculate a retardation in maturation of the renal function in male infants compared to females as is known for the development of the pulmonary function [52].

All parameters evaluated in this study are regularly used to monitor either electrolyte homeostasis or bone mineralization. Practices among units vary greatly, measurements may be performed at later age and greater intervals and not standardized or in combination, leading to inconsistent results and handling. An explanation for the inconsistency in results of other studies could be the underestimation of the effects of inter-relationships between various co-variates. In our opinion, these associations can only be discovered with standardized repeated measurements taking into account other clinical factors. To our knowledge, this is the first study evaluating changes in biochemical parameters of the calcium-phosphorus homeostasis based on standardized repeated measurements and daily changes in nutritional intake in a mixed model linear regression analysis including also clinical factors.

Our data show that standardized repeated measurements of blood and urine samples can provide useful information with regard to the Ca-P homeostasis. This does not result in a clear advice for nutritional intake. Nevertheless, this study is a first step and its importance lies in the description and quantification of changes in a more “physiological way” that will further enable us to develop new guidelines to improve bone mineral status in preterm infants. Notwithstanding, we confirmed the relationship between the intake of protein and phosphorus, and demonstrated the effect of renal immaturity and gender. Thus, a second step could be, to relate the current results to bone mineralization and provide recommendations for nutritional intake and a third step to develop a concept of target values for biochemical parameter so that these can be used to monitor nutritional intake to achieve optimal bone mineralization in daily practice.

This study had several limitations. The mixed model analysis assumes that the effects of the different variables are linear which has not been proven yet. In addition, the biochemical parameters may have been influenced by factors that were not taken into account in our analysis. Daily sampling of biochemical parameter would have been optimal, but this was judged unethical regarding the amount of blood volume needed. Nevertheless, measurements were performed in a standardized manner and therefore provided a good reflection of changes in blood and urine concentrations for the complete study period. Further, in comparison to other studies, both groups had relatively high daily intakes. This may partly explain the small variations in biochemical parameters. This study only investigated the biochemical parameters during the first 14 days of life. Maturational changes in renal function may alter the results; however, repeated measurements will indicate these changes and thereby can be used as guide for optimal supplementation of minerals.

## 5. Conclusions

In conclusion, standardized repeated measurements showed that biochemical parameters of Ca-P homeostasis seemed to be affected by nutritional intake of calcium and phosphorus as well as protein, while immaturity of kidneys was related to an increase in urinary excretion of minerals irrespective of nutritional intake. Further studies are needed to define target values to stabilize electrolyte balances and improve bone mineralization taking into account nutritional intake and gestational age of the patient.

## Figures and Tables

**Table 1:** Patient characteristics, morbidity, medication, and nutritional characteristics.

	Low (*n* = 40)	High (*n* = 30)	*p*-Value
**Characteristics**			
Birth weight, grams; median (IQR)	948 (772–1225)	939 (776–1163)	0.85
<1000 gram, *n* (%)	22 (55.0)	16 (53.3)	0.89
Gestational age, median (IQR)	28.2 (26.5–29.6)	27.8 (26.1–29.4)	0.76
SGA, *n* (%)	8 (20.0)	4 (13.3)	0.46
Male, *n* (%)	19 (47.5)	15 (50.0)	0.84
Singletons, *n* (%)	28 (70.0)	16 (53.3)	0.15
Cesarean section, *n* (%)	18 (45.0)	20 (66.7)	0.07
Apgar score (5 min), median (IQR)	7.0 (6.3–9.0)	7.5 (7.0–8.0)	0.71
Apgar score (5 min) <7, *n* (%)	10 (25.0%)	6 (20.0%)	0.62
**Morbidity**			
Sepsis, *n* (%)	8 (20.0%)	9 (30.0%)	0.33
NEC ≥ stage 2, *n* (%)	1 (2.5%)	2 (6.7%)	0.39
IVH Grade 3–4 *n* (%)	5 (12.5)	3 (10)	0.75
**Medication**			
Caffeïne; *n* (%)	39 (97.5%)	28 (93.3%)	0.39
Furosemide, *n* (%)	3 (7.5%)	4 (13.3%)	0.42
Corticosteroids	3 (7.5)	1 (3.3)	0.46
Sedation, *n* (%)	8 (20.0%)	9 (30.0%)	0.33
**Nutritional characteristics**			
PN, days, median (IQR)	11.0 (9.0–14.0)	12.0 (10.0–14.0)	0.10
150 mL/kg enteral, study day, median (IQR)	12.0 (9.8–17.0)	13.0 (10.5–20.0)	0.59
Start day of BMF, median (IQR)	11 (11.0–13.0)	7.9 (5.0–10.0)	0.00
Human milk in mL/kg/day *, median (IQR)	50.9 (24.0–82.2)	30.0 (8.5–54.6)	0.01
Formula in mL/kg/day *, median (IQR)	0.0 (0.0–0.2)	1.1 (0.1–7.3)	0.00
**Nutritional intake**			
Ca (total) W1, mmol/kg; median (IQR)	10.7 (9.9–12.0)	13.1 (11.1–14.6)	0.00
Ca (total) W2, mmol/kg; median (IQR)	16.4 (12.9–17.7)	21.7 (15.3–24.4)	0.00
P (total) W1, mmol/kg; median (IQR)	10.8 (9.3–12.4	12.3 (11.1–14.2)	0.00
P (total) W2, mmol/kg; median (IQR)	16.4 (12.9–19.6)	18.2 (16.0–22.1)	0.02
Prot (total) W1, grams/kg; median (IQR)	18.6 (15.9–21.1)	20.0 (16.9–23.4)	0.16
Prot (total) W2, grams/kg; median (IQR)	23.2 (21.0–26.6)	27.0 (24.1–30.6)	0.00

Low: no enteral supplementation of human milk before Day 11; High: standard protocol: enteral supplementation of human milk if intake was ≥50 mL/day; IQR: Interquartile range; SGA: small for gestational age: <p10; Sepsis: >72 h postnatally and positive blood culture, prevalence within the first 14 days; NEC: necrotizing enterocolitis according to Bell stage [36], prevalence within the first 14 days; IVH: Intraventricular hemorrhage (grade according to Papile) [37]; PN: parenteral nutrition; BMF: breast milk fortifier; *: during the intervention period; Ca: calcium; P: phosphorus; Prot: protein; W1: Week 1; W2: Week 2.

**Table 2:** Mixed Model analysis: Effect of nutritional intake and clinical characteristics on biochemical parameter.

Dependant Variable	Covariates	Estimate	95% CI	*p*-Value
Serum Calcium	Total intake of Ca (mmol/kg/day)	0.004	−0.046–0.054	0.89
	Total intake of P (mmol/kg/day)	−0.036	−0.073–0.002	0.06
	Enteral intake of P (%)	0.001	−0.000–0.001	0.06
	Intake of protein (grams/kg /day)	0.107	0.075–0.139	0.00
	Gestational age (weeks)	0.027	0.013–0.042	0.00
	Singleton (yes)	0.081	0.021–0.140	0.01
	Sepsis (yes)	−0.092	−0.167–−0.019	0.02
	Sedation (days)	−0.007	−0.016–0.001	0.07
Serum Phosphorus	Total intake of Ca (mmol/kg/day)	0.0345	−0.0473–0.1164	0.41
	Total intake of P (mmol/kg/day)	0.1252	0.0586–0.1918	0.00
	Enteral intake of Ca (%)	0.0035	0.0023–0.0048	0.00
	Intake of protein (grams/kg/day)	−0.1274	−0.1825–−0.0723	0.00
	Birth weight (grams)	0.0004	0.0002–0.0006	0.00
	Gestational age (weeks)	−0.0479	−0.0701–−0.0258	0.00
	Gender (boy)	−0.0698	−0.1493–0.096	0.08
	Caffeine (days)	−0.0215	−0.0354–−0.0075	0.00
	Furosemide (days)	−0.1116	−0.2029–−0.0203	0.02
Urine Calcium	Total intake of Ca (mmol/kg/day)	0.35	0.01–0.70	0.05
	Total intake of P (mmol/kg/day)	−0.01	−0.29–0.27	0.94
	Enteral intake of Ca (%)	−0.02	−0.02–−0.01	0.00
	Intake of protein (grams/kg/day)	0.36	0.12–0.61	0.00
	Cesarean section (yes)	0.65	0.32–0.98	0.00
Urine Phosphorus	Total intake of Ca (mmol/kg/day)	−0.05	−1.56–1.45	0.94
	Total intake of P (mmol/kg/day)	3.18	2.06–4.30	0.00
	Enteral intake of P (%)	0.07	0.04–0.09	0.00
	Intake of protein (grams/kg/day)	−1.18	−2.20–−0.16	0.02
	Gestational age (weeks)	−0.71	−1.09–−0.33	0.00
	Gender (boy)	1.88	0.26–3.50	0.02
	Caffeine (days)	−0.29	−0.54–−0.01	0.04
Tubular reabsorption of P	Total intake of Ca (mmol/kg/day)	3.10	0.160–6.04	0.04
	Total intake of P (mmol/kg/day)	−6.21	−8.78–−3.65	0.00
	Enteral intake of P (%)	−0.09	−0.15–−0.03	0.01
	Gestational age (weeks)	3.05	1.92–4.17	0.00
	Gender (boy)	−4.60	−9.22–0.01	0.05
	Cesarean section (yes)	−5.12	−9.95–−0.29	0.04
	Sepsis (yes)	−6.78	−12.72–−0.85	0.03
	Furosemide (days)	4.75	−0.53–10.03	0.07
Alkaline Phosphatase	Total intake of Ca (mmol/kg/day)	−44.94	−69.51–−20.37	0.00
	Total intake of P (mmol/kg/day)	23.64	4.14–43.14	0.02
	Enteral intake of Ca (%)	2.07	1.69–2.45	0.00
	Intake of protein (grams/kg/day)	30.54	14.08–47.01	0.00
	Gestational age (weeks)	−20.71	−30.37–−11.05	0.00
	Postnatal steroids (days)	−23.86	−44.29–−3.43	0.02
Urine Ca/Crea ratio	Total intake of Ca (mmol/kg/day)	0.138	−0.292–0.568	0.53
	Total intake of P (mmol/kg/day)	0.139	−0.204–0.481	0.43
	Enteral intake of Ca (%)	−0.023	−0.029–−0.016	0.00
	Intake of protein (grams/kg/day)	0.497	0.206–0.787	0.01
	Sepsis (yes)	0.584	0.003–1.166	0.05
Urine P/Crea ratio	Total intake of Ca (mmol/kg/day)	−1.10	−2.51–0.31	0.12
	Total intake of P (mmol/kg/day)	4.01	2.97–5.05	0.00
	Enteral intake of P (%)	0.06	0.04–0.08	0.00
	Intake of protein (grams/kg/day)	−0.81	−1.76–0.14	0.10
	Gestational age (weeks)	−0.94	−1.32–−0.55	0.00
	Gender (boy)	2.31	0.72–3.89	0.01
	Sepsis (yes)	1.72	−0.24–3.68	0.09
	Caffeine (days)	−0.30	−0.56–−0.03	0.03

sCa: serum calcium (mmol/L); uCa: urine calcium (mmol/L); sP: serum phosphorus (mmol/L); uP: urine phosphorus (mmol/L); TrP: tubular reabsorption of phosphorus (%); ALP: Alkaline phosphatase (U/L); uCa/Crea ratio: urine calcium/creatinin ratio (mmol/mmol); uP/Crea ratio: urine phosphorus/creatinine ratio (mmol/mmol); 95% CI: 95% confidence interval; Co-variables initially included: daily nutritional intake of calcium, phosphorus, and protein, the enteral amount of calcium and phosphorus intake, caesarian section, multiple births, birth weight, gestational age, gender, necrotizing enterocolitis, sepsis, caffeine, furosemide, steroids and sedation.

## Appendix A

**Table A1:** Standard parenteral nutritional intake.

	Day 1	Day 2	Day 3	Day 4
Fluid mL/kg/day	80	100	125	150
CH grams/kg/day	8	9.6	11.7	13.8
AA grams/kg/day	0.75	1.5	2.25	3
Lipids grams/kg/day	1	2	3	3
EQ Kcal/kg/day	44	62	82	94
Calcium mmol/kg/day	0.75	1.5	2.25	3.00
Phosphorus mmol/kg/day	0.48	0.96	1.44	1.92

Parenteral nutritional intake based on standardized parenteral solutions [35,53]. For infants below 1000 grams, amino acids were additionally added according to current recommendations [12]. Amino acid solution: Primene (Baxter, the Netherlands); Lipid emulsion including vitamins: Clinoleic (20%; Baxter, The Netherlands) or SMOFlipid 20% (Fresenius Kabi; The Netherlands); CH: carbohydrates, AA: amino acids, EQ: energy quotient.

**Table A2:** Cohort characteristics of all patients included in the Early Supplementation Study.

Characteristics	Group ENS A (*n* = 40)	Group ENS B (*n* = 32)	Group C (*n* = 37)
GA, weeks; med (IQR)	28.2 (25.7–30.1)	28.3 (26.5–30.7)	27.9 (26.1–29.7)
Birth weight, grams; med (IQR)	967 (753–1245)	1012 (847–1199)	1006 (771–1220)
SGA; *n* (%)	9 (23)	8 (25)	6 (16)
Male; *n* (%)	21 (53)	20 (63)	18 (49)
Singletons; *n* (%)	25 (63)	24 (74)	25 (68)
Antenatal Steroids compl.; *n* (%)	36 (90)	31 (97)	31 (86)
Cesarean section; *n* (%)	19 (48)	20 (63)	25 (68)
Apgar score (5 min); med (IQR)	7.5 (6.3–9.0)	8.0 (7.0–9.0)	7.0 (7.0–8.0)
Apgar score (5 min) <7; *n* (%)	10 (25)	5 (16)	8 (22)
Mortality; *n* (%)	6 (15)	3 (9)	7 (19)
Morbidity			
IRDS	24 (60)	19 (59)	23 (62)
Days of MV; med (IQR)	1.5 (0.0–4.8)	1.0 (0.0–4.0)	1.0 (0.0–7.0)
Days of N-CPAP; med (IQR)	18.0 (6.5–38.8)	28.0 (7.0–40.8)	16.0 (6.0–36.5)
CLD; *n* (%)	12 (30)	14 (44)	10 (27)
PDA; *n* (%)	20 (50)	20 (63)	21 (57)
Ductal ligation; *n* (%)	4 (10)	2 (6.3)	1 (3)
IVH grade ≤ 2; *n* (%)	15 (38)	5 (16)	5 (14)
IVH grade ≥ 3; *n* (%)	2 (5)	7 (21)	4 (11)
Sepsis; *n* (%)	13 (33)	10 (32)	14 (38)
NEC; *n* (%)	4 (10)	5 (16)	3 (8)
Bell stage 2; *n*	2	3	1
Bell stage 3; *n*	2	2	2
Laparotomy; *n*	2	1	2
ROP; *n* (%)	4 (10)	1 (3)	5 (14)
ROP grade ≥ 3	1	0	1
Medication			
Caffeine; *n* (%)	38 (95)	30 (94)	33 (90)
Furosemide; *n* (%)	11 (28)	10 (31)	7 (19)
Diuretics (maintenance); *n* (%)	3 (8)	0	3 (8)
Corticosteroids; *n* (%)	1 (3)	2 (6)	4 (11)
Sedation; *n* (%)	13 (33)	11 (34)	15 (41)
Nutritional characteristics			
Days of PN; med (IQR)	10.0 (8.0–13.0)	10.5 (9.0–14.8)	10.5 (8.3–21.0)
120 mL/kg enteral, day; med (IQR)	9.0 (7.0–12.5)	9.0 (8.0–13.0)	9.0 (7.2–14.8)
150 mL/kg enteral, day; med (IQR)	12.0 (9.0–17.0)	11.0 (10.0–17.0)	12.0 (10.0–20.0)
Start day of BMF; med (IQR)	11.0 (11.0–12.7)	12.0 (11.0–14.0)	6.0 (4.0–8.0)

ENS A: donor milk in addition to mother’s own milk (MOM) and no supplements with enteral feeding until Day 10; ENS B: preterm formula in addition to MOM and no supplements with enteral feeding until Day 10; Group C: preterm formula in addition to MOM and fortifier if intake ≥50 mL/day; med: median; IQR: inter quartile range; GA: gestational age; SGA: small for gestational age according to Fenton et al. [54]; IRDS: infant respiratory distress syndrome; MV: mechanical ventilation; N-CPAP: nasal continuous positive airway pressure; CLD: chronic lung disease defined as oxygen dependency at 36 weeks gestational age; PDA: patent ductus arteriosus with need for treatment; IVH: intra-ventricular hemorrhage; Sepsis: >72 h postnatally and positive blood culture; NEC: necrotizing enterocolitis with staging according to Bell [36]; ROP: retinopathy of prematurity; sedation: morfine and/or midazolam >24 h; PN: parenteral nutrition; BMF: breast milk fortifier.

**Table A3:** Nutritional intake of calcium and phosphorus by route of administration.

Nutritional Intake (mmol/kg/Week)	Low (*n* = 40) Median (IQR)	High (*n* = 30) Median (IQR)	*p*-Value
Ca (total) W1	10.7 (9.9–12.0)	13.1 (11.1–14.6)	0.00
Ca (total) W2	16.4 (12.9–17.7)	21.7 (15.3–24.4)	0.00
Ca (enteral) W1	1.7 (1.1–2.2)	3.3 (1.1–5.7)	0.00
Ca (enteral) W2	10.8 (6.2–16.0)	17.5 (2.3–22.8)	0.07
Ca (enteral suppl) W1	0.0 (0.0–0.0)	0.0 (0.0–0.0)	1.0
Ca (enteral suppl) W2	0.0 (0.0–0.0)	0.0 (0.0–0.0)	0.22
Ca (PN) W1	9.4 (8.0–10.2)	9.8 (7.9–11.5)	0.34
Ca (PN) W2	3.1 (0.7–7.9)	5.1 ( 1.9–10.9)	0.14
Ca (PNsuppl) W1	0.0 (0.0–0.0)	0.0 (0.0–0.0)	1.0
Ca (PNsuppl) W2	0.0 (0.0–0.0)	0.0 (0.0–0.0)	0.74
P (total) W1	10.8 (9.2–12.4)	12.3 (11.1–14.2)	0.00
P (total) W2	16.4 (12.9–19.6)	18.9 (16.0–22.1)	0.02
P (enteral) W1	1.0 (0.6–1.4)	2.1 (0.7–3.9)	0.00
P (enteral) W2	8.1 (5.0–12.0)	10.4 (1.7–17.2)	0.08
P (enteral suppl) W1	0.0 (0.0–0.0)	0.0 (0.0–0.9)	0.03
P (enteral suppl) W2	0.0 (0.0–0.0)	0.0 (0.0–1.4)	0.33
P (PN) W1	7.5 (6.4–8.2)	7.8 (6.3–9.2)	0.34
P (PN) W2	2.5 (0.6–6.3)	4.1 (1.5–8.7)	0.14
P (PN suppl) W1	2.4 (0.5–3.8)	1.8 (0.0–2.7)	0.16
P (PN suppl) W2	2.3 (0.0–4.2)	0.7 (0.0–4.3)	0.30

Low: no enteral supplementation of human milk before Day 11; High: standard protocol: enteral supplementation of human milk if intake was ≥50 mL/day; IQR: inter quartile range; Ca: calcium; P: phosphorus; total: som of all nutritional intake; enteral: enteral intake including standard fortification; enteral suppl: additional enteral supplementation; PN: parenteral intake; PN suppl: additional parenteral supplementation; W1: week 1; W2: week 2.

**Table A4:** Daily measurements of biochemical parameter of the calcium and phosphorus homeostasis.

		Day 1	Day 3	Day 5	Day 7	Day 10	Day 14
Serum Ca (mmol/L)	Low	2.2 (2.0–2.4)	2.4(2.2–2.5)	2.5 (2.4–2.7)	2.6 (2.4–2.7)	2.5 (2.4–2.7)	2.6 (2.4–2.8)
	High	2.2 (2.0–2.4)	2.4 (2.3–2.6)	2.5 (2.4–2.7)	2.4 (2.3–2.6)	2.5 (2.3–2.7)	2.6 (2.5–2.8)
	*p-Value*	*0.94*	*0.17*	*0.34*	*0.15*	*0.69*	*0.17*
Urine Ca (mmol/L)	Low	1.3 (1.0–1.9)	2.1 (1.5–3.4)	2.6 (2.0–3.9)	2.7 (1.6–3.8)	2.0 (1.6–3.3)	1.8 (1.4–3.5)
	High	1.5 (1.2–1.7)	3.3 (2.0–4.7)	3.1 (2.3–5.8)	2.5 (1.8–3.8)	2.7 (2.0–3.4)	2.3 (1.6–3.4)
	*p-Value*	*0.66*	*0.03*	*0.06*	*0.84*	*0.21*	*0.50*
Serum P (mmol/L)	Low	1.8 (1.7–2.2)	1.8 (1.5–2.0)	1.7 (1.5–2.0)	1.9 (1.8–2.2)	2.0 (1.8–2.2)	2.3 (2.1–2.4)
	High	2.1 (1.8–2.3)	1.8 (1.5–2.0)	1.6 (1.3–2.2)	2.1 (1.8–2.4)	2.1 (2.0–2.4)	2.2 (2.0–2.3)
	*p-Value*	*0.22*	*0.88*	*0.71*	*0.21*	*0.08*	*0.16*
Urine P (mmol/L)	Low	1.7 (0.3–3.8)	2.1 (0.7–5.5)	1.3 (0.7–3.5)	4.1 (0.7–6.8)	5.4 (2.2–8.2)	10.4 (6.8–18.4)
	High	2.3 (0.2–4.6)	2.6 (0.9–4.1)	3.1 (1.6–6.9)	5.4 (3.5–9.8)	7.4 (4.5–13.7)	9.5 (5.5–16.4)
	*p-Value*	*0.68*	*0.98*	*0.04*	*0.02*	*0.04*	*0.46*
Tubular reabsorption of P (%)	Low	94.0 (76.8–98.2)	86.5 (76.1–95.3)	92.2 (83.8–96.9)	83.1 (72.4–96.3)	80.8 (68.8–92.7)	75.9 (64.9–85.2)
	High	85.3 (76.4–98.1)	92.7 (73.3–95.7)	88.1 (80.8–95.1)	79.4 (51.9–87.6)	76.6 (59.1–83.2)	67.4 (53.9–82.4)
	*p-Value*	*0.59*	*0.62*	*0.21*	*0.08*	*0.23*	*0.36*
Alkaline Phosphatase (U/L)	Low	167.0 (138.0–236.0)	213.0 (182.0–291.0)	263.5 (202.3–368.5)	329 (261.3–455.0)	379 (311.0–503.0)	423.0 (301.8–506.0)
	High	203 (146.5–232.8)	244.0 (174.3–270.8)	275.5 (206.3–307.0)	304.5 (249.3–375.0)	342.0 (213.8–414.3)	380.0 (281.8–509.8)
	*p-Value*	*0.76*	*0.94*	*0.76*	*0.22*	*0.10*	*0.66*
uCa/Crea ratio (mmol/mmol)	Low	2.1 (1.3–3.5)	2.5 (1.8–3.1)	3.6 (2.3–5.2)	3.8 (2.3–6.2)	2.9 (1.8–4.1)	2.3 (1.6–3.7)
	High	1.7 (1.4–3.7)	2.7 (2.0–6.1)	4.0 (2.9–6.3)	3.0 (2.1–5.4)	3.8 (2.4–5.0)	3.4 (1.9–4.8)
	*p-Value*	*0.98*	*0.30*	*0.35*	*0.49*	*0.21*	*0.14*
uP/Crea ratio (mmol/mmol)	Low	1.6 (0.5–5.9)	2.8 (1.0–6.8)	1.9 (0.8–5.4)	5.1 (1.1–9.3)	7.2 (2.5–12.3)	11.2 (6.2–18.6)
	High	3.8 (0.5–6.7)	1.8 (1.1–5.8)	3.5 (1.6–6.1)	7.7 (5.0–10.9)	9.4 (7.0–16.5)	14.5 (9.3–18.7)
	*p-Value*	*0.64*	*0.72*	*0.15*	*0.14*	*0.06*	*0.73*

All data are presented as median and interquartile range; sCa: serum calcium (mmol/L); uCa: urine calcium (mmol/L); sP: serum phosphorus (mmol/L); uP: urine phosphorus (mmol/L); TrP: tubular reabsorption of phosphorus (%); ALP: Alkaline phosphatase (U/L); uCa/Crea ration: urine calcium/creatinine ration (mmol/mmol); uP/Cre ratio (mmol/mmol).
